# Correlation between skeletal muscle fiber type and free amino acid levels in Japanese Black steers

**DOI:** 10.1111/asj.13185

**Published:** 2019-02-27

**Authors:** Daisuke Mashima, Yoshiaki Oka, Takafumi Gotoh, Shozo Tomonaga, Shoko Sawano, Mako Nakamura, Ryuichi Tatsumi, Wataru Mizunoya

**Affiliations:** ^1^ Department of Bioresource Sciences Faculty of Agriculture Kyushu University Fukuoka Japan; ^2^ Department of Agricultural Sciences and Natural Resources Faculty of Agriculture Kagoshima University Kagoshima Japan; ^3^ Division of Applied Biosciences Graduate School of Agriculture Kyoto University Kyoto Japan

**Keywords:** beef, fiber type, free amino acid, myosin heavy chain, skeletal muscle

## Abstract

Free amino acids are important components of tastants and flavor precursors in meat. To clarify the correlation between muscle fiber type and free amino acids, we determined the concentrations of various free amino acids and dipeptides in samples of different muscle tissues (*n* = 21), collected from 26‐month‐old Japanese Black steers (*n* = 3) at 2 days postmortem. The proportions of the myosin heavy chain (MyHC), slow (MyHC1) and fast (MyHC2) isoforms were determined by sodium dodecyl sulfate–polyacrylamide gel electrophoresis (SDS–PAGE). The contents of free amino acids and dipeptides were measured by high performance liquid chromatography (HPLC). The MyHC isoform composition varied among the tissue samples. The MyHC1 proportion ranged from 6.9% ± 3.9% to 83.3% ± 16.7%. We confirmed that there was a strong positive correlation between MyHC1 composition and total free amino acid concentrations, including those for two dipeptides. Among the 31 measured free amino acids and dipeptides, 11 showed significant positive correlations and five showed significant negative correlations with MyHC1 composition. These results suggest that a high MyHC1 content induces high free amino acid contents in bovine muscles possibly because of greater oxidative metabolism. This high level of free amino acids could contribute to the intense flavor of meat that is rich in slow‐twitch fibers.

## 1 INTRODUCTION

Skeletal muscle tissues are composed of slow‐twitch (type 1) and fast‐twitch (type 2) muscle fibers. Metabolically, slow‐twitch fibers have abundant mitochondria and myoglobin and rely on oxidative metabolism, whereas fast‐twitch fibers have less mitochondria and myoglobin and mainly rely on the glycolytic pathway. Myosin heavy chain (MyHC) is a predominant and key component of skeletal muscle proteins and is used as a marker protein of muscle fiber type. To date, four predominant MyHC isoforms have been identified in adult mammalian skeletal muscles: MyHC1, 2A, 2X, and 2B (Pette & Staron, 2000). Generally, each muscle fiber (muscle cell) expresses only one MyHC isoform. MyHC1 is expressed in type 1 muscle fibers. Type 2 fibers are subdivided into types 2A, 2X, and 2B in order of increasing contraction speed. In these fibers, MyHC2A, 2X, and 2B isoforms are preferentially expressed in type 2A, 2X, and 2B muscle fibers, respectively. Among these MyHC2 isoforms, MyHC2B appears to be specific to small mammals (Liu et al., 2009) and marsupials (Zhong, Lucas, & Hoh, 2008). In bovine muscles, type 2B fibers are not expressed in the limb or trunk muscles (Toniolo et al., 2005).

Muscle fiber composition is thought to affect the color, pH, water holding capacity, tenderness, and nutritional value of meat (Choi & Kim, 2009), but the relationship between the free amino acid content and muscle fiber composition of meat is not fully understood. Free amino acids contained in meat are important flavor precursors (Toldrá, Flores, & Sanz, 1997). For instance, amino acids are converted to amines by decarboxylation (Hernández‐Jover, Izquierdo‐Pulido, Veciana‐Nogués, & Vidal‐Carou, 1996) and are essential for Maillard reactions that produce numerous volatile products (Mottram, 1998). Moreover, the free amino acid level is important for taste (Kato, Rhue, & Nishimura, 1989; Nishimura, Ra Rhue, Okitani, & Kato, 1988). If the free amino acid level of meat can be determined, it could be used to assess meat quality.

We hypothesized that slow‐twitch fibers would contain more free amino acids than fast‐twitch fibers because of the slow‐twitch fiber mitochondria content. Enzymes for the tricarboxylic acid (TCA) cycle are abundant in mitochondria and many free amino acids are metabolized from substrates in this cycle. The aim of this study was to determine the relationship between muscle fiber type and free amino acid content, using 21 samples of different skeletal muscle tissues from Japanese Black steers.

## 2 MATERIALS AND METHODS

### 2.1 Animals and tissue collection

Three Japanese Black steers (26 months old) raised in Kuju Agricultural Research Center, Kyushu University were used. The steers were cared for and slaughtered according to Guidelines for Animal Experiments in the Faculty of Agriculture of Kyushu University and to laws of the Japanese Government (Law No. 105, Notification No. 6). The steers were raised in a pen with group feeding using the slightly modified standard feeding system for the production of marbled beef (Albrecht et al., 2011; Gotoh et al., 2009). The steers were slaughtered in an approved slaughterhouse and the carcasses were kept at 0°C for 48 hr. Then, they were transported to a meat processing facility at room temperature for 2 hr and processed within 4 hr. We collected samples of 21 different muscle tissues from all over the carcass (Table 1) and stored at −80°C. While removing obvious intermuscular fats and connective tissues, each muscle tissue was ground to a powder with a mortar and pestle, cooled with liquid nitrogen, and stored at −80°C again until required for analysis.

**Table 1:** Myosin heavy chain (MyHC) composition of various muscle samples from Japanese Black steers (%)

Muscle	MyHC1	MyHC2	Standard deviation	Minimum MyHC1	Maximum MyHC1
Rectus femoris	6.9	93.1	3.9	2.4	9.3
Semitendinosus	8.8	91.2	5.7	3.2	14.6
Semimembranosus	12.5	87.5	2.2	10.9	15.0
Longissimus thoracis	14.8	85.2	8.3	7.1	23.6
Biceps femoris (distal portion)	15.5	84.5	1.5	13.8	16.6
Vastus lateralis	16.5	83.5	2.7	14.9	19.6
Latissimus dorsi	24.1	75.9	8.9	18.6	34.4
Psoas major	30.4	69.6	4.5	25.2	33.8
Rhomboideus thoracis	37.0	63.0	11.1	24.3	45.3
Splenius capitis	43.1	56.9	28.7	10.1	62.8
Biceps femoris (proximal portion)	46.1	53.9	17.8	35.6	66.6
Spinalis and semispinalis	54.5	45.5	10.8	45.5	66.5
Subscapularis	55.1	44.9	8.8	46.7	64.3
Trapezius	56.6	43.4	26.6	27.6	80.0
External oblique	57.1	42.9	2.4	55.0	59.7
Seratus cervicis	60.6	39.4	23.3	46.1	87.4
Iliacus	67.0	33.0	1.0	66.2	68.1
Infraspinous	71.8	28.2	12.6	60.9	85.6
Flexor digitorum superficialis	71.8	28.2	21.3	54.5	95.5
Gluteus accesorius	78.6	21.4	7.7	70.1	85.2
Serratus ventralis	83.3	16.7	12.2	69.1	90.6

*Note*. The data are from three Japanese Black steers.

Minimum and maximum MyHC1 values are across the three cattle.

### 2.2 MyHC isoform content

A motor‐driven small pestle was used to homogenize each sample of muscle (~50 mg) in a SDS solution (10% SDS, 40 mM dithiothreitol (DTT), 5 mM ethylenediamine tetraacetic acid (EDTA), and 0.1 M Tris‐HCl buffer (pH 8.0)). The SDS solution contained protease inhibitor cocktail for use with mammalian cell and tissue extracts (Nacalai Tesque, Inc., Kyoto, Japan) in a 1:100 ratio. The sample homogenates were heated in boiling water for 3 min. Total protein concentrations were assayed using the Pierce BCA Protein Assay Kit (Thermo Fisher Scientific, Waltham, MA, USA), with bovine serum albumin as a standard. The samples were diluted in 2 × sample buffer (100 mM DTT, 4.0% SDS, 0.16 M Tris‐HCl (pH 6.8), 43% glycerol, and 0.2% bromophenol blue) and dH2O to give final protein concentrations of 20 ng/μl in 1 ×  sample buffer. These protein samples were subjected to high‐resolution SDS–PAGE to assess the MyHC isoform composition, as described in detail previously (Mizunoya, Wakamatsu, Tatsumi, & Ikeuchi, 2008). The gel was 8% acrylamide (acrylamide/bisacrylamide ratio = 99:1) and contained 35% (v/v) glycerol. After loading the samples (100 ng protein), electrophoresis was performed at a constant voltage of 140 V for 22 hr at 4°C. The gels were stained with Silver Stain Kanto III (Kanto Chemical Co., Inc., Tokyo, Japan) and dried. The bands were captured on an image scanner, and the relative contents of MyHC isoforms were quantified by densitometry using ImageJ 1.34s software (Rasband W, National Institutes of Health, USA). MyHC isoforms were identified according to their different migration rates (MyHC1 > 2).

### 2.3 Measurements of amino acids

Free amino acids in the muscle samples were determined by HPLC (Pico‐Tag™; Waters) in accordance with a previous method (Rubio, 2003). The tissue samples were homogenized in an ice‐cold 0.2 M perchloric acid solution, containing 0.01 mM EDTA and left for deproteinization on ice for 30 min. Then, the tissue homogenates were centrifuged at 20,000 × *g* for 15 min at 0°C. The supernatants were filtered through 0.20‐μm filters (Millipore, Bedford, USA), and each 20‐μl muscle sample solution was dried under reduced pressure. The residues were dissolved in 10 μl of 1 M sodium acetate–methanol–triethylamine (2:2:1, v/v/v), dried under reduced pressure, and then redissolved in 20 μl of methanol–distilled water–triethylamine–phenylisothiocyanate (7:1:1:1, v/v/v/v), which is a derivatization solution. After reaction of the phenylisothiocyanate with the amino groups at room temperature for 20 min, the samples were dried again, dissolved in 200 μl of Pico Tag diluent, and filtered through a Syringe‐driven Filter Unit (Millex‐LG; Millipore) to remove any solids. A standard amino acid mixture (Type AN II and Type B; Wako Pure Chemical Industries, Ltd.) was applied using the same methods. The derivatized samples and standards were analyzed by HPLC with equilibration using buffer A (70 mM sodium acetate (pH 6.45 with 10% acetic acid) and acetonitrile, 975:25 v/v) and eluted with a linear gradient of buffer B (water–acetonitrile–methanol, 40:45:15 v/v/v) at a flow rate of 1 ml/min at 46°C. The concentrations of free amino acids were determined from the absorbance values at 254 nm. The muscle amino acid concentrations are expressed as micromoles per gram of wet tissue.

### 2.4 Statistics

Results are expressed as means ± *SD*. Pearson correlation coefficients were calculated using Excel 2004 (Microsoft) to determine the relationship between MyHC1 composition and free amino acid content. We used a two‐tailed *t* test, and significance was set at *p *< 0.05.

## 3 RESULTS AND DISCUSSION

**Figure 1:**
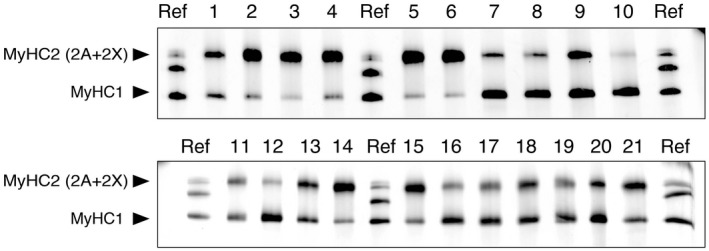
Separation of MyHC isoforms by sodium dodecyl sulfate–polyacrylamide gel electrophoresis (SDS–PAGE) for representative bovine muscle samples. Lanes: 1, biceps femoris (proximal portion); 2, biceps femoris (distal portion); 3, rectus femoris; 4, vastus lateralis; 5, semimembranosus; 6, semitendinosus; 7, iliacus; 8, gluteus accessorius; 9, flexor digitorum superficialis; 10, serratus ventralis; 11, serratus cervicis; 12, trapezius; 13, rhomboideus thoracis; 14, latissimus dorsi; 15, longissimus thoracis; 16, spinalis and semispinalis; 17, splenius capitis; 18, subscapularis; 19, infraspinous; 20, external oblique; 21, psoas major; and ref, a mix of rat extensor digitorum longus muscles and soleus sample that was used as the four MyHC isoform reference (migration rate is MyHC1 > 2B > 2X > 2A)

We measured the muscle fiber type compositions in 21 steers’ muscle tissue samples (Figure 1 and Table 1). Separation of MyHC2A and MyHC2X was not sufficient under the present electrophoretic conditions, and we used the sum of MyHC2A and MyHC2X as the MyHC2 content. Different MyHC isoform compositions (MyHC1 and MyHC2) were observed in the various muscle tissues. The lowest MyHC1 proportion was observed in the rectus femoris (6.9% ± 3.9%) and the highest MyHC1 proportion was observed in the serratus ventralis (83.3% ± 16.7%). Our results agreed with those of previous studies showing that the rectus femoris muscle contains predominantly fast‐twitch fibers in cattle (Kirchofer, Calkins, & Gwartney, 2002) and pigs (Suzuki, Watanabe, Konno, & Ohwada, 1999). However, the serratus ventralis muscle has previously been classified as an intermediate muscle, which means that its slow‐ and fast‐twitch fiber composition is balanced (Kirchofer et al., 2002; Robe & Xiong, 1994). Because the fiber‐type composition can differ even within the same muscle tissue according to the muscle portion (e.g., cranial, middle, or caudal) (Suzuki et al., 1999), the serratus ventralis muscle could have a high slow‐twitch fiber composition in some portions. In our experiments, the MyHC1 composition of the proximal portion of the biceps femoris muscle was about three times that in the distal portion (Table 1).

**Figure 2:**
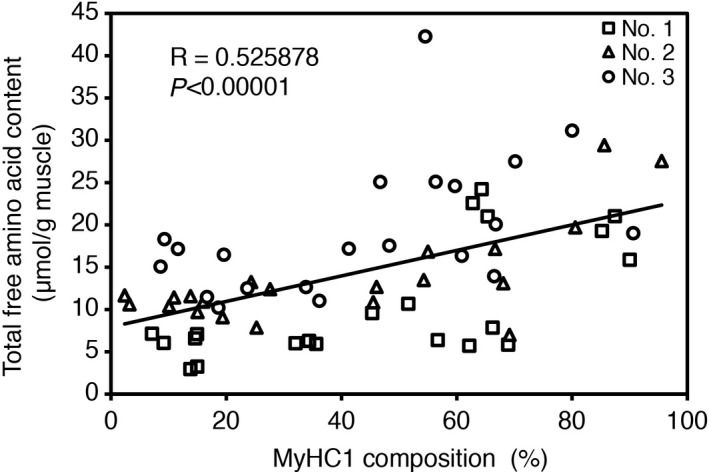
Correlation between total free amino acid contents and the proportion of MyHC1 in samples from 21 different muscle tissues in Japanese Black steers. Different symbols indicate different animals (*n* = 3, labeled as Nos. 1–3)

We investigated the correlation between the proportion of MyHC1 and the total free amino acid and dipeptide contents and found a strong positive correlation (*p *< 0.00001) (Figure 2 and Table 2). This indicates that an increase in slow‐twitch fiber content induces an increase in the total free amino acid content. This correlation could be related to meat flavor through the effects of amino acids as taste enhancers or precursors of aroma compounds (Toldrá et al., 1997). In fact, in a tasting panel evaluation of lamb, redder meat, which is rich in slow‐twitch fibers, was classed as having a more intense flavor than whiter meat, which is rich in fast‐twitch fibers (Valin, Touraille, Vigneron, & Ashmore, 1982).

**Table 2:** Correlations between the proportions of MyHC1 (%) and free amino acid contents in muscle samples from Japanese Black steers

Amino acids and dipeptides	Correlation coefficients (*r*)	Significance	Mean (μmol/g)	Minimum (μmol/g)	Maximum (μmol/g)	Threshold (mM)(Schiffman et al., 1981)
Alanine	0.458	*p* < 0.01	3.740	0.830	7.408	16.2
Arginine	0.051	NS	0.259	0.091	0.563	1.20
Asparagine	0.107	NS	0.113	0.022	0.390	1.62
Aspartic acid	−0.252	*p* < 0.05	0.093	0.012	0.226	0.18
β‐alanine	0.369	*p* < 0.01	0.099	0.011	0.321	
Citrulline	−0.027	NS	0.025	ND	0.066	
Cystathionine	0.098	NS	0.003	0.00014	0.017	
γ‐aminobutyric acid	0.111	NS	0.053	ND	0.257	
Glutamic acid	0.164	NS	5.203	0.603	22.859	0.06
Glutamine	0.639	*p* < 0.01	1.127	0.154	2.950	9.8
Glycine	0.033	NS	0.664	0.196	1.817	30.9
Histidine	0.254	*p* < 0.05	0.153	0.035	0.537	1.23
1‐methyl Histidine	0.305	*p* < 0.05	0.003	ND	0.019	
3‐methyl Histidine	0.414	*p* < 0.01	0.029	ND	0.221	
Hydroxyproline	0.428	*p* < 0.01	0.048	0.008	0.176	
Isoleucine	−0.192	NS	0.169	0.041	0.402	7.41
Leucine	−0.165	NS	0.351	0.106	0.870	6.45
Lysine	0.003	NS	0.355	0.055	0.973	0.71
Methionine	−0.081	NS	0.077	0.017	0.216	3.72
Ornithine	0.605	*p* < 0.01	0.206	0.009	0.991	
Phenylalanine	−0.211	NS	0.142	0.030	0.377	6.61
Phosphoserine	−0.507	*p* < 0.01	0.572	0.082	1.966	
Proline	0.099	NS	0.179	0.059	0.392	15.1
Serine	0.303	*p* < 0.05	0.326	0.114	0.291	20.9
Taurine	0.270	*p* < 0.05	4.881	0.552	23.861	
Threonine	0.188	NS	0.201	0.064	0.398	25.7
Tryptophan	0.531	*p* < 0.01	0.928	0.021	4.768	2.29
Tyrosine	−0.261	*p* < 0.05	0.127	0.032	0.299	
Valine	−0.153	NS	0.278	0.003	0.621	4.16
Anserine	−0.383	*p* < 0.01	1.104	ND	2.876	
Carnosine	−0.559	*p* < 0.01	8.291	0.012	18.508	
Total amino acids and dipeptides	0.526	*p* < 0.01	14.5	3.0	42.3	

*Note*. Cysteine was not detected.

Mean, minimum, and maximum values were calculated from 63 values (21 muscle samples × 3 animals).

NS, not significant; ND, not detected.

The correlations between the MyHC1 proportion and the free amino acids are summarized in Table 2. Among the 31 free amino acids and dipeptides, 11 showed significant positive correlations and five showed significant negative correlations with MyHC1 composition. Marked positive correlations were observed for alanine, β‐alanine, glutamine, 3‐methylhistidine, hydroxyproline, ornithine, and tryptophan (*p *< 0.01). Glutamine is a key precursor of α‐ketoglutarate, which is an intermediate in the TCA cycle. Furthermore, histidine, which positively correlated with MyHC1 (*p *< 0.05), enhances metabolic reaction rates in the TCA cycle (Shimizu, 2007). This correlation is consistent with the high oxidative metabolism expected in slow‐twitch fibers. Contrastingly, marked negative correlations were observed for phosphoserine, anserine, and carnosine (*p *< 0.01), which suggests that these compounds are abundant in fast‐twitch fibers. We expected to find negative correlations for anserine and carnosine because white muscles, which are rich in fast‐twitch fibers, generally have greater anserine and carnosine concentrations than red muscles, which are rich in slow‐twitch fibers. For example, the concentrations of these dipeptides in chicken breast muscle (white muscle) are six times those in chicken thigh muscle (red muscle) (Crush, 1970). Anserine and carnosine concentrations in porcine longissimus dorsi (white muscle) are 1.5 times those in vastus intermedius (red muscle) (Mei, Cromwell, Crum, & Decker, 1998).

Most free amino acids stimulate taste and can modify the palatability of foods depending on the concentrations at which they are present (Schiffman, Hornack, & Reilly, 1979; Schiffman, Sennewald, & Gagnon, 1981). Thus, it is important to know whether the concentrations of free amino acids exceed the taste detection thresholds. In Table 2, we have referred the taste thresholds in the right‐hand column. Concentrations of free amino acids in meat are expressed in micromoles per gram of wet tissue, and solution‐based concentrations of these substances could not be determined without water content information. Here, we simply replaced the wet tissue mass with water volume. The concentrations of glutamic acid in bovine muscles exceeded the threshold in all samples, even at the minimum value; however, this amino acid did not show a significant correlation to fiber type. The samples with the maximum contents of aspartic acid and lysine exceeded the taste thresholds. Though lysine did not show a significant correlation with muscle fiber type, aspartic acid, which tastes flat, sour, and slightly bitter, showed a significant negative correlation with the MyHC1 proportion and may contribute to the taste of meat with high fast‐twitch fiber content. Overall, the contents of most of the free amino acids in meat were below the taste thresholds.

It should be noted that the actual taste response will be more complicated because of the effects of taste enhancers. Interestingly, purine nucleotides, such as inosine monophosphate (IMP), considerably potentiate the taste responses of free amino acids. Nelson et al. showed that stimulation of mouse fungiform papillae with various amino acids and IMP enhanced the responses of the chorda tympani induced by the amino acids (Nelson et al., 2002). Therefore, purine nucleotide contents in meat should be examined in future. During postmortem meat aging, adenosine triphosphate‐related compounds degrade and generate products such as IMP and hypoxanthine. It has been suggested that the contents of purine nucleotides and their metabolites differ according to the muscle fiber composition in porcine muscles (Chikuni et al., 2013), and high levels of IMP and adenosine triphosphate degradation products have been found in fast‐twitch fiber predominant muscle tissues. Therefore, the relationship between nucleotide levels and muscle fiber types should be investigated in detail in various meats in the future.

In conclusion, we found that there was a strong positive correlation between the MyHC1 proportion and total free amino acid concentrations, including those of two dipeptides. Among the 31 free amino acids and dipeptides, 11 showed significant positive correlations and five showed significant negative correlations with MyHC1 proportion. These results suggest that high levels of slow MyHC1 induce high free amino acid contents in bovine muscles possibly because of greater oxidative metabolism. The high levels of free amino acids could contribute to the intense flavor of meat that is rich in slow‐twitch fibers.

## ACKNOWLEDGMENTS

This research was supported by a research grant from the Society for Research on Umami Taste. This research was also supported by the Japan Society for the Promotion of Science (JSPS) KAKENHI #26712023 to WM; 17H03908 to WM and RT; 17K07807 to WM and SS. The cost of English proofreading service was supported by a Research Grant for Young Investigators of Faculty of Agriculture, Kyushu University.
